# Araport: the Arabidopsis Information Portal

**DOI:** 10.1093/nar/gku1200

**Published:** 2014-11-20

**Authors:** Vivek Krishnakumar, Matthew R. Hanlon, Sergio Contrino, Erik S. Ferlanti, Svetlana Karamycheva, Maria Kim, Benjamin D. Rosen, Chia-Yi Cheng, Walter Moreira, Stephen A. Mock, Joseph Stubbs, Julie M. Sullivan, Konstantinos Krampis, Jason R. Miller, Gos Micklem, Matthew Vaughn, Christopher D. Town

**Affiliations:** 1 Plant Genomics, J. Craig Venter Institute, Rockville, MD 20850, USA; 2 Texas Advanced Computing Center, The University of Texas, Austin, TX 78758, USA; 3 Cambridge Systems Biology Centre, University of Cambridge, Cambridge CB2 1QR, UK

## Abstract

The Arabidopsis Information Portal (https://www.araport.org) is a new online resource for plant biology research. It houses the *Arabidopsis thaliana* genome sequence and associated annotation. It was conceived as a framework that allows the research community to develop and release ‘modules’ that integrate, analyze and visualize Arabidopsis data that may reside at remote sites. The current implementation provides an indexed database of core genomic information. These data are made available through feature-rich web applications that provide search, data mining, and genome browser functionality, and also by bulk download and web services. Araport uses software from the InterMine and JBrowse projects to expose curated data from TAIR, GO, BAR, EBI, UniProt, PubMed and EPIC CoGe. The site also hosts ‘science apps,’ developed as prototypes for community modules that use dynamic web pages to present data obtained on-demand from third-party servers via RESTful web services. Designed for sustainability, the Arabidopsis Information Portal strategy exploits existing scientific computing infrastructure, adopts a practical mixture of data integration technologies and encourages collaborative enhancement of the resource by its user community.

## INTRODUCTION

The flowering plant *Arabidopsis thaliana* is a model organism for molecular, cellular and systems biology. Arabidopsis offers the favorable characteristics of short lifecycle, prolific seed production and genomic transformation efficiency. The *A. thaliana* Col-0 ecotype offers a nearly complete genome sequence assembly and high-quality gene annotation, along with many informatic and experimental resources. Other ecotypes and mutant lines, available in seed form, have been genotypically and phenotypically characterized. For over a decade, The Arabidopsis Information Resource (TAIR) served as the collector, curator and provider of pertinent information centered on the reference Col-0 genome. In 2009, the Arabidopsis community was faced with a growing number of online resources and potential loss of its central organizing resource, TAIR. After a series of workshops, members of the Arabidopsis research community published a white paper calling for a new Arabidopsis Information Portal (AIP) (1). The portal would assume responsibility for curation of the genome sequence and gene annotation. Intensive human curation of gene function, literature association etc. is not part of AIP's funded mandate. Rather, it will use an extensible architecture that will permit growth through contributions of community-curated data-centric modules embracing a wide range of data types. With NSF and BBSRC funding, we presented in 2014 a first functional release of Araport (ICAR 2014 proceedings).

Following its termination of funding and in parallel with the development of the AIP, TAIR began to require subscription fees in 2014, allowing it to continue its gene-centric data curation services (ICAR 2014 proceedings) that will complement the work of AIP. The AIP project does, and will continue to, incorporate all the pertinent data that TAIR makes public on a time-delayed schedule. Future Araport releases could act as a gateway by providing access to additional data for registered users of TAIR and other subscription-based services.

The AIP is freely available online at https://www.araport.org. It offers two core web applications: ThaleMine for data mining and JBrowse for genome browsing. It hosts an embryonic collection of ‘science apps’ that should serve as prototypes for community-contributed modules. It offers a reproducible and extensible framework, based on data federation and community-generated content, that should allow it to serve the growing and changing Arabidopsis fields of omics research.

## CORE FUNCTIONALITY

**Figure 1.**
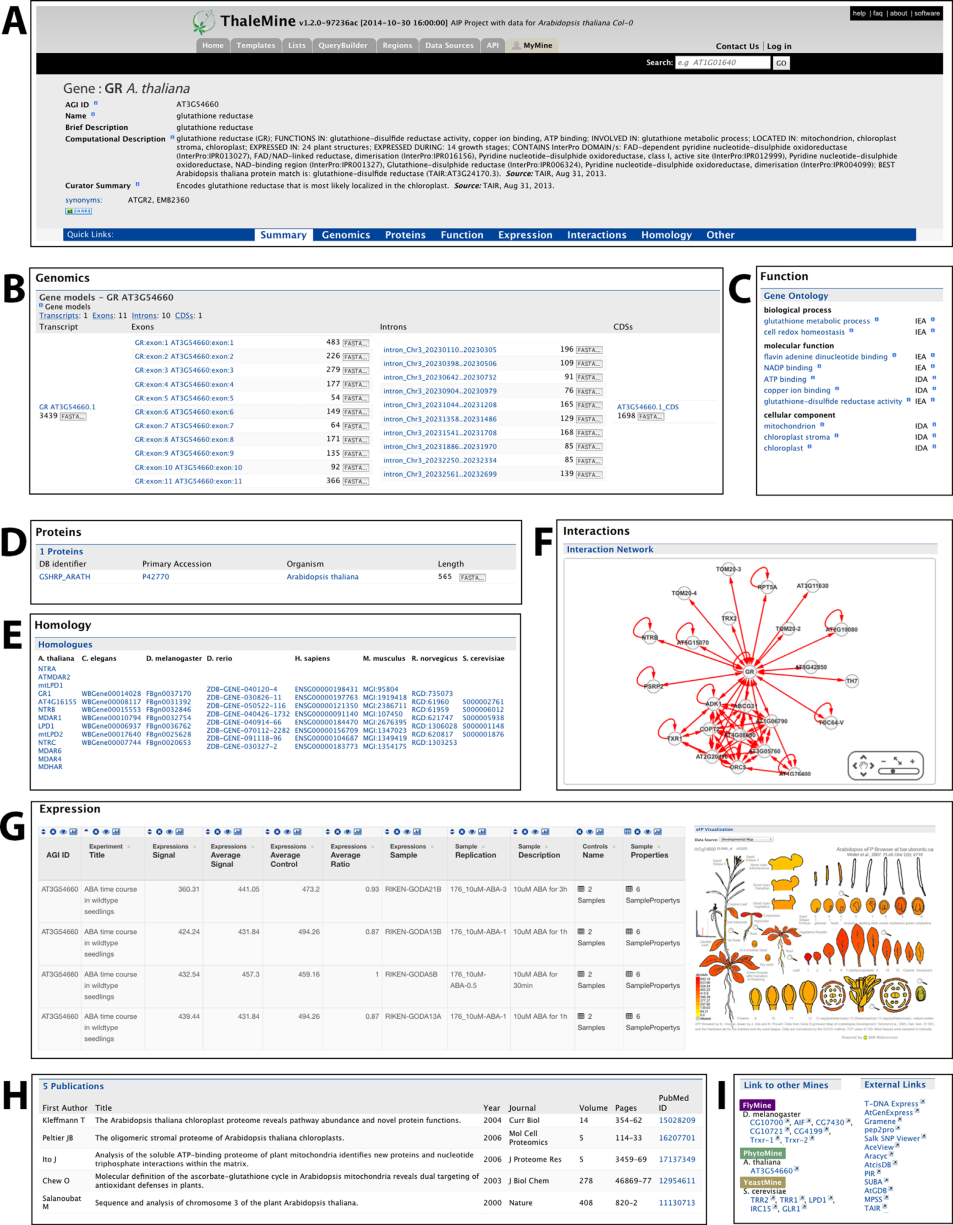
A Gene report page showing: (**A**) attributes like standard gene locus identifier, symbols/synonyms, TAIR curator summary, confidence rating, etc. Other useful information is segregated into the aspects, as follows: (**B**) ‘Genomics’ showing gene structure information; (**C**) ‘Function’ displaying the Gene Ontology annotation; (**D**) ‘Proteins’ with links to the relevant protein records populated with data from UniProt; (**E**) ‘Homology’ listing the computed orthologs and paralogs across a diverse set of species; (**F**) ‘Interactions’ showing a visual representation of the genes’ physical/genetic interactions (tabular format is also available); (**G**) ‘Expression’ reporting gene expression levels based on AtGenExpress project data, also visualized using an embedded eFP view; (**H**) ‘Publications’ reports papers associated with the current gene; (**I**) ‘Links’ to other InterMine databases where homologs of the current gene exist, and external links to several important Arabidopsis and plant genomics data providers.

The core of AIP is a database of basic genomic data within a data mining web application called ThaleMine. ThaleMine is a customized instance of the InterMine software (2), which provides parsers for many data sources, rapid retrieval of indexed data, dynamic table presentation, saved and shared list functionality, and web services (3) support. Currently, ThaleMine includes the TAIR 10 Col-0 sequence and annotation dataset (4). Assimilation of other data types (polymorphisms, phenotypes, stocks, etc.) is in progress. ThaleMine integrates data from UniProtKB (5), PubMed (6), Bio-Analytic Resource (BAR) (7), PANTHER (8), Sequence Ontology (9) and Gene Ontology (10). ThaleMine links to sources including PhytoMine implemented by Phytozome (11), FlyMine (12) and YeastMine (13) (Table 1). ThaleMine organizes 33 602 genes (including transposable element and pseudogenes), 41 671 transcripts, 31 189 transposable elements, 47 698 proteins, 16 127 publications, 125 expression studies, 6365 protein domains, 197 625 interactions and 5323 gene ontology terms. AIP loaded ThaleMine with an extensible set of template-based queries, which provide simple web interfaces to common kinds of searches (Table 2). The application supports free-text search as well as structured queries. For instance, searches can return lists of loci with specified attributes within the *A. thaliana* Col-0 reference genome sequence. List analysis is facilitated by widgets that generate column-wise statistics, identify over-represented terms or publication links or compare lists by set-union and set-intersection. Capability to drill down by locus is facilitated by report pages for genes, transcripts and proteins. These pages display gene structure, functional annotation, protein domains, protein interactions, associated publications, expression patterns, co-expressed genes, orthologs in other species and links to other resources. Locus-specific pages also display an embedded BAR Electronic Fluorescent Pictograph (eFP) viewer (14) (Figure 1). The ThaleMine User Guide (https://www.araport.org/thalemine/user-guide) has more details.

**Table 1.** Summary of the datasets used to populate ThaleMine and their corresponding sources

Dataset	Data source
Genome sequence and annotation	TAIR (version 10; 8/31/13)
Protein sequence and properties	UniprotKB
Protein interactions	Bio-Analytic Resource (BAR)
Affymetrix expression data	AtGenExpress via BAR
Electronic Fluorescent Pictographs	Bio-Analytic Resource
Publications	UniprotKB and NCBI Entrez
Orthologs	PANTHER and PhytoMine

**Table 2.** List of commonly executed searches provided as ‘Template searches’

Input (accepts wildcard characters)	Output (reported as a table, exportable in different file formats)
Gene (identifier/alias)	Related protein identifiers
	List of interactors
	Set of homologous genes
	Expression values
	Publications referencing this gene
	FASTA sequences of the CDS
	FASTA sequence of the proteins
	FASTA sequences of the 5′/3′ UTRs
Protein (identifier/domain)	Related gene identifiers
	Publications referencing this protein
Ontology term	List of genes

**Figure 2.**
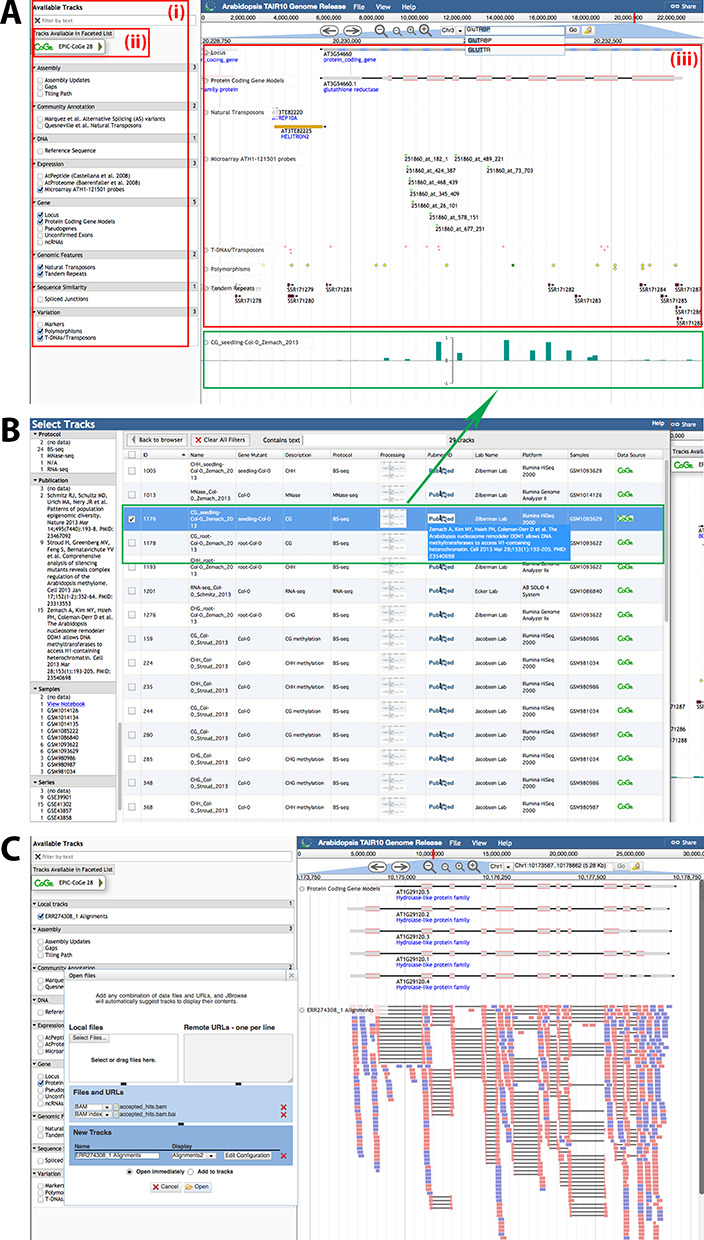
(**A**) (i) List of tracks available via the hierarchical track selector; (ii) a link to access the faceted track selector; (iii) track display panel showing the currently selected tracks for viewing (green inset: track chosen from the faceted selector). (**B**) Faceted track selector offers a search and filtering interface to enable choosing tracks based on the experiment metadata. (**C**) File upload capability ensures privacy to view ones own datasets alongside the Arabidopsis genome, and flexibility to work with large datasets such as short read alignments.

AIP hosts a customized instance of JBrowse (15) that, like InterMine, is actively supported by the GMOD consortium (http://www.gmod.org). The browser integrates gene structure, various kinds of evidence and genomic attributes, expression data, and more. Below the reference sequence axis, the browser presents a stack of ‘tracks’ representing, for example, the TAIR 10 gene models. The browser currently incorporates 20 tracks, including datasets such as pseudogenes, expression, non-coding RNAs, epigenetics and T-DNA insertion lines, which are presented to the user via the hierarchical track selector (Figure 2A). Additionally, 28 more tracks (not in the hierarchical selector) are obtained on-demand from EPIC CoGe (16) via web services, which are cataloged within the faceted track selector (Figure 2B). We modified JBrowse to enable the hierarchical and faceted track selectors to co-exist. This allows users to select from our 20 standard tracks and also our 28 metadata-rich tracks organized by user-set facets of their metadata. The browser also offers bulk download of interval-specific track data in common file formats (FASTA, GFF3, BED). Similarly, it can integrate user-provided datasets, either local or hosted on third party servers (i.e. displaying tracks of read mappings without transmitting entire files, by exploiting indexed BAM files) without data transmission to AIP servers (Figure 2C). While searching or scrolling, the page URL is dynamically updated with sufficient information to fully reproduce the display. This feature enables collaboration by URL sharing between remote users.

## EXTENSIBLE FUNCTIONALITY

The AIP design relies on data federation and software modularity for scalability and sustainability. The AIP module concept consists of a visual front end to an externally provided database or computational resource. AIP encourages the Arabidopsis research community to contribute modules as a means of disseminating their results. AIP provides the framework for developing, hosting and integrating modules as well as software and training materials. The AIP software development kit (SDK) provides boilerplate code (as an example it deploys a ‘Hello World’ app, the classic first task for programmers), serving as a starting point for developers. The SDK was built with node.js (http://nodejs.org), grunt (http://gruntjs.com) and yeoman (http://yeoman.io) software. Using this SDK, AIP has developed and hosts several demonstration modules. One is a BLAST (17) module offering homology search and sequence alignment visualization. Another provides graphical and tabular viewers for *Arabidopsis* protein–protein interactions, for which the data are obtained on demand from EBI IntAct web services (18) and the visualization relies on BioJS (19) and Cytoscape (20) open-source software. AIP science apps can access web services directly or through the AIP middleware. They can access AIP-hosted web services that expose the data, metadata and indexes associated with ThaleMine and JBrowse at AIP.

AIP hosts community information. This includes notices of news regarding the AIP project, postings syndicated from GARNet (http://www.garnetcommunity.org.uk), conferences/events and job openings. AIP has a forum for posting, and answering, questions to AIP staff and the community. AIP offers files for bulk download including the latest public TAIR release of whole genome sequence (FASTA), gene structure annotation (GFF3) and other supporting datasets.

## ARCHITECTURE

With the goal of providing the Arabidopsis research community with a sustainable resource, Araport is designed to be scalable and reproducible. For scalability, its organizing principle is federation. AIP hosts the middleware and services that enable the development and deployment of community-contributed science apps. As enablers, AIP hosts the two data-centric applications, ThaleMine and JBrowse, which provide data services, visualization tools and optional launch points for third-party modules. For reproducibility, AIP maintains public repositories hosted on GitHub (https://www.github.com), tracking the data, configuration and software code that are required to recreate the Araport website. We already rebuild parts of Araport (e.g. ThaleMine) automatically from scripts in the repository. We aim to automatically deploy the complete site at two locations (JCVI, TACC) and possibly others.

The AIP core applications, ThaleMine and JBrowse, were both implemented by customizing open-source GMOD software. The core applications represent a mixture of the data federation and data hosting approaches to website development. Both applications use federation to obtain data on demand from remote sites. ThaleMine displays links to orthologs and eFP expression pattern images, both dependent on run-time data requests to third-party web services. ThaleMine and JBrowse not only consume but also expose web services. These web services have RESTful interfaces, as first described by Fielding (21). They are exposed through an AIP mediation layer in an effort to increase their uniformity, stability and ease of use (e.g. by precluding cross-origin resource sharing (CORS) security restrictions). Both ThaleMine and JBrowse also rely on AIP-hosted data, currently ∼150 GB, sufficient to provide rapid displays and search responses. Hosted data include genome sequence; IDs and functional descriptions of genes, transcripts, proteins, domains and ontological terms; and metadata used to identify genome browser tracks. A portion of ThaleMine data is obtained from TAIR on a delayed-release schedule. Other data are downloaded from FTP sites and web services shortly before deploying the applications. A Chado database (22), with Tripal modules (23), is under construction. It will act as a data warehouse in support of future application deployments.

The Araport software employs a strongly decoupled three-tier design in which front end presentation software communicates through a middle layer to back end databases. This architecture was adopted to promote site sustainability by enabling future re-engineering of each tier independently. The AIP presentation layer uses HTML5 (http://www.w3.org/TR/html5/), CSS (http://www.w3.org/Style/CSS/), JavaScript, AJAX and jQuery (http://jquery.com) delivered via HTTPS to provide a responsive and secure experience to clients using modern web browsers on any platform. AIP's middle layer employs the Apache Tomcat web server, InterMine software, the Drupal content management system, the iPlant Collaborative-developed Agave API platform (24) and OAuth2 (http://tools.ietf.org/html/rfc6749) authentication. On the back end, AIP employs PostgreSQL (http://www.postgresql.org) databases and the iPlant Data Store (25) for data persistence. Modules of AIP communicate with databases via AIP's mediating layer that provides authentication and other services (logging, throttling, load balancing, data transformation, caching). Communications between front-to-middle layers and middle-to-back layers are implemented as web services. These exchange JSON-formatted data over the HTTPS protocol using RESTful URL standards being developed and documented at AIP.

## FUTURE DIRECTIONS

As TAIR's NSF funding ended, responsibility for the *A. thaliana* Col-0 reference genome and annotation passed to AIP. We plan to update the standard ‘TAIR10′ sequence and annotation with releases labeled ‘AIP11’, ‘AIP12’, etc. We are implementing an automated re-annotation pipeline that combines publicly available RNA-seq reads, their Trinity *de novo* and reference-guided assemblies (26) and PASA annotation comparison (27). One goal is to delineate tissue-specific transcript isoforms. We will solicit expert community review to be implemented with Tripal and WebApollo (28) software.

Araport is designed to respond to a recent whitepaper (1) that called for a sustainable community portal whose growth would depend on modular contributions from the Arabidopsis research community. Now entering its second year of funding, Araport provides two fully functional web applications for browsing and data mining *A. thaliana* genomics data. More importantly for growth, the AIP staff is working with the community to extend its documentation, middleware and SDKs that enable third-party module development and dissemination. By early November 2014, AIP will have hosted its first workshop for community developers. The workshop is expected to generate about a dozen modules addressing research areas including expression, regulation and epigenetics. Each module will offer dynamic web pages providing visualization of remote data that is accessed on demand through RESTful web services. This and other workshops should enhance the repertoire of available modules, increase community involvement, lower the technical hurdles for contribution, and eventually enable increasing levels of integration and sophistication of module design. AIP will continue to enhance its presentation layer to aid users in the discovery, use and combination of modules. AIP will integrate third-party modules into its core applications and simultaneously promote the integration of core application web services and visualizations within third-party modules. This federated model of data sharing and software development should help AIP continuously adapt to the omics data explosion and the resulting needs of the broader Arabidopsis user community.
